# miR-34c-3p Regulates Protein Kinase A Activity Independent of cAMP by Dicing *prkar2b* Transcripts in Theileria annulata-Infected Leukocytes

**DOI:** 10.1128/msphere.00526-22

**Published:** 2023-02-27

**Authors:** Malak Haidar, Shahin Tajeri, Laurence Momeux, Tobias Mourier, Fathia Ben-Rached, Sara Mfarrej, Zineb Rchiad, Arnab Pain, Gordon Langsley

**Affiliations:** a Pathogen Genomics Laboratory, Bioscience Program, Biological and Environmental Sciences and Engineering (BESE) Division, King Abdullah University of Science and Technology (KAUST), Thuwal, Kingdom of Saudi Arabia; b Université Paris Cité, CNRS, INSERM, Institut Cochin, Paris, France; c Institute of Biological Sciences (ISSB), Faculty of Medical Sciences, Mohammed VI Polytechnic University (UM6P), Ben Guerir, Morocco; d International Institute for Zoonosis Control, GI-CoRE, Hokkaido University, Sapporo, Japan; e Institute of Parasitology and Tropical Veterinary Medicine, Freie Universität Berlin, Berlin, Germany; f de Duve Institute, Université Catholique de Louvain, Brussels, Belgium; University at Buffalo

**Keywords:** PKA regulatory subunit, PRKAR2B, *Plasmodium falciparum*, *Theileria*, miR-34c-3p, microRNA

## Abstract

MicroRNAs (miRNAs) are small noncoding RNAs that can play critical roles in regulating various cellular processes, including during many parasitic infections. Here, we report a regulatory role for miR-34c-3p in cAMP-independent regulation of host cell protein kinase A (PKA) activity in *Theileria annulata*-infected bovine leukocytes. We identified *prkar2b* (cAMP-dependent protein kinase A type II-beta regulatory subunit) as a novel miR-34c-3p target gene and demonstrate how infection-induced upregulation of miR-34c-3p repressed PRKAR2B expression to increase PKA activity. As a result, the disseminating tumorlike phenotype of *T. annulata*-transformed macrophages is enhanced. Finally, we extend our observations to Plasmodium falciparum-parasitized red blood cells, where infection-induced augmentation in miR-34c-3p levels led to a drop in the amount of *prkar2b* mRNA and increased PKA activity. Collectively, our findings represent a novel cAMP-independent way of regulating host cell PKA activity in infections by *Theileria* and *Plasmodium* parasites.

**IMPORTANCE** Small microRNA levels are altered in many diseases, including those caused by parasites. Here, we describe how infection by two important animal and human parasites, Theileria annulata and Plasmodium falciparum, induce changes in infected host cell miR-34c-3p levels to regulate host cell PKA kinase activity by targeting mammalian *prkar2b*. Infection-induced changes in miR-34c-3p levels provide a novel epigenetic mechanism for regulating host cell PKA activity independent of fluxes in cAMP to both aggravate tumor dissemination and improve parasite fitness.

## INTRODUCTION

MicroRNAs (miRNAs) are a class of small noncoding RNAs and are key regulators in several biological processes, ranging from development and metabolism to apoptosis and signaling pathways (1, 2). Indeed, their profiles are altered in many human diseases, particularly in cancer (3, 4), making them a major focus of research. The up- or downregulation of miRNAs can cause drastic changes to gene expression due to the fact that miRNAs have up to hundreds of potential mRNA targets (5). Changes to gene expression can lead to malignant transformation, especially if miRNA targets include genes that regulate cell homeostasis, proliferation, cell cycle progression, adhesion, or dissemination (6).

Posttranscriptional control of gene expression by miRNAs is also increasingly recognized as a central part of host/pathogen interactions. The role of miRNAs in bacterial (7, 8), viral (9), parasitic helminth (10), and protozoan infections is now well established. *Plasmodium* and *Theileria* parasites are obligate intracellular protozoa of the phylum Apicomplexa. They are closely related and have similar life cycles, including infection of a vertebrate intermediate host and an arthropod definitive host. Apicomplexan parasites are able to alter the global gene expression patterns of their respective host cells by interfering with signaling cascades to neutralize host defenses (11) and improve their capacity to infect, proliferate, and disseminate. Apicomplexa also manipulate their host cell’s miRNomes to their own benefit (12–14). For example, we have shown that miR-126-5p directly targets and suppresses JNK interacting protein 2 (JIP-2). As a result, JNK translocates to the host cell nucleus and phosphorylates c-Jun, which transactivates AP-1-driven transcription of *mmp9* to promote the tumor dissemination of *Theileria*-transformed macrophages (15). Moreover, miR-155 is also induced by infection to suppress de-etiolated homolog 1 expression, which diminishes c-Jun ubiquitination (16). An increase in c-Jun levels leads to an augmentation in *bic* (pri-miR-155) transcripts that contain miR-155, explaining how a positive feedback loop contributes to the growth and survival of *Theileria*-infected leukocytes (16). Furthermore, exosomes and their miRNA cargo have been found to play an important role in the manipulation of the host cell phenotype and the pathobiology of both *Theileria* and *Plasmodium* infections (17, 18). Moreover, miRNAs have been detected in extracellular vesicles (EV) derived from Plasmodium falciparum-infected red blood cells (iRBCs), where they are bound to AGO2, forming functional complexes (18). In addition, P. falciparum-infected RBCs further accumulate microvesicles containing AGO2 and miRNA released from infected RBCs (19).

Herein, we demonstrate that by targeting *prkar2b* coding for the protein kinase A type II-beta regulatory subunit, miR-34c-3p regulates mammalian PKA activity independently of cAMP (cyclic AMP) in *Theileria*-infected macrophages. We have previously shown that attenuated Theileria annulata-infected macrophages used as live vaccines against tropical theileriosis have diminished PKA activity and consequently impaired dissemination potential (20). We now show that attenuated macrophages have lower levels of miR-34c-3p and correspondingly higher levels of PRKAR2B, which leads to diminished PKA activity that contributes to the attenuated dissemination phenotype.

Erythrocyte infection by P. falciparum also leads to an increase in miR-34c-3p levels (18), so we asked whether this led to an increase in infected erythrocyte PKA activity. We confirmed that infection of red blood cells by P. falciparum leads to increased amounts of miR-34c-3p and demonstrated that inhibition of miR-34c-3p binding to its cognate seed sequence raised *prkar2b* transcript levels and reduced infected red blood cell PKA kinase activity. So, in two different host-parasite combinations, infection-induced increases in the miR-34c-3p lead to a reduction in *prkar2b* transcripts and an increase in host PKA activity independent of fluxes in cAMP.

## RESULTS

### miR-34c-3p is differentially expressed in *T. annulata*-infected leukocytes.

**FIG 1:**
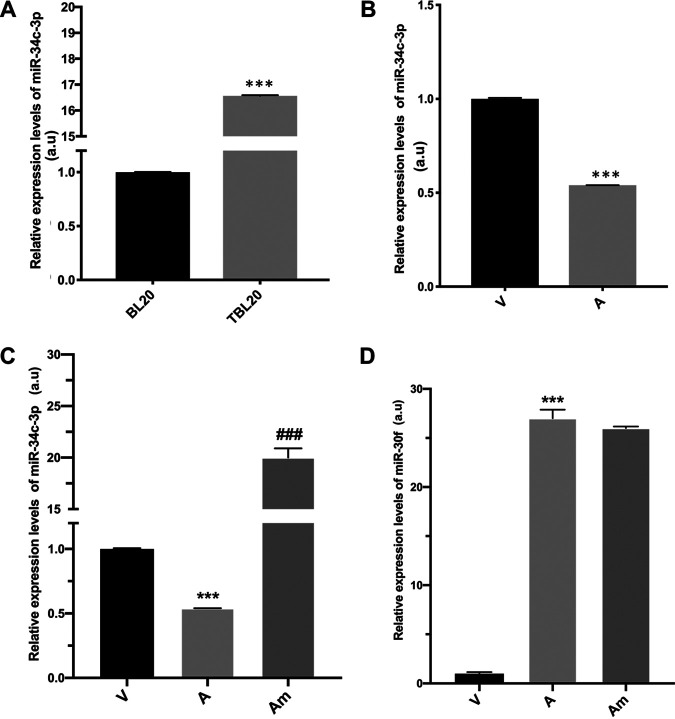
miR-34c-3p is differentially expressed in *T. annulata*-infected leukocytes. (A) miR-34c-3p levels in uninfected B lymphocytes (BL20) increase when BL20 are infected with *T. annulata* (TBL20). (B) Levels of miR-34c-3p are high in virulent macrophages and decrease in attenuated macrophages. (C) miR-34c-3p levels in attenuated macrophages increase following their transfection with miR-34c-3p mimic. (D) Endogenous levels of miR-30f are low in virulent (V) macrophages and high in attenuated (A) macrophages. Transfection of attenuated macrophages with the miR-34c-3p mimic (Am) did not significantly change miR-30f levels, demonstrating the specificity of the miR-34c-3p mimic. Data are represented as mean ± SEM. *n *=* *3. ***, *P < *0.001 compared to virulent macrophages; ###, *P < *0.001 compared to attenuated macrophages. miR-34c-3p levels were estimated using qPCR.

*T. annulata*-transformed virulent macrophages disseminate in infected animals, causing a disease called tropical theileriosis. However, upon long-term *in vitro* passaging, their capacity to disseminate becomes attenuated, and they are used as live vaccines to control disease (21). We focused on miR-34c, as its expression is upregulated upon infection of B cells (Fig. 1A). Comparing virulent (V) and attenuated (A) infected macrophages reveals that miR-34c levels are significantly downregulated upon the attenuation of infected macrophage dissemination (Fig. 1B). We artificially increased the miR-34c-3p levels of attenuated macrophages by transfecting them with a miR-34c-3p mimic (Fig. 1C). As a negative control, we chose miR-30f, whose endogenous levels are inversely correlated with those of miR-34c-3p by being low in virulent (V) and high in attenuated (A) macrophages (Fig. 1D). Transfection of the mimic did not increase the miR-30f levels in attenuated macrophages, confirming its specificity for miR-34c-3p.

### Changes in miR-34c-3p levels influence the dissemination of *T. annulata*-transformed macrophages.

**FIG 2:**
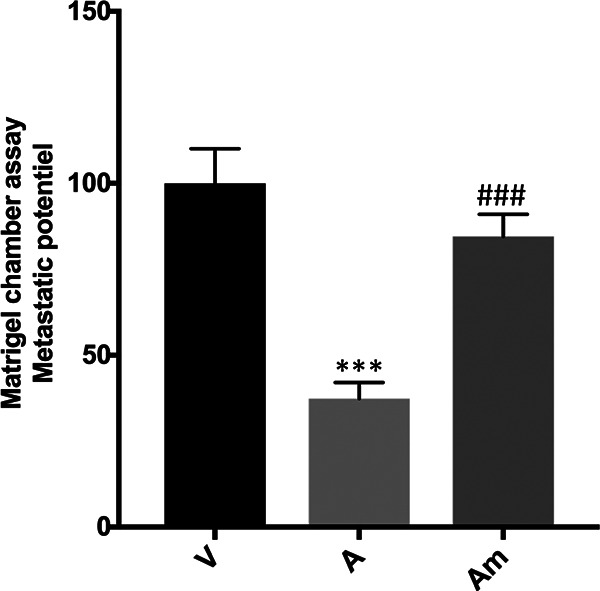
Changes in miR-34c-3p levels impact the dissemination potential of *T. annulata*-transformed macrophages. The ability of virulent (V) macrophages to traverse Matrigel is greater than that of attenuated (A) macrophages. Transfection of attenuated macrophages with the miR-34c-3p mimic (Am) restored Matrigel traversal to virulent macrophage levels. Data are represented as mean ± SEM. ***, *P* *<* 0.001 compared to virulent macrophages; ###, *P* < 0.001 compared to attenuated macrophages.

Virulent macrophages display a high capacity to traverse Matrigel compared to attenuated macrophages (20). Since levels of miR-34c-3p are downregulated in attenuated macrophages, its positive regulatory effect on the capacity of *T. annulata*-transformed macrophages to traverse Matrigel was examined. Attenuated macrophages were transfected with the miR-34c mimic, and their Matrigel traversal capacities were examined (Fig. 2). The increase in miR-34c-3p levels restored the Matrigel traversal of the attenuated macrophages to levels equivalent to those of virulent macrophages (Fig. 2). This demonstrates that infection-induced increase in miR-34c-3p levels contributes to aggressive dissemination of *T. annulata*-transformed macrophages.

### *prkar2b* is a direct target gene of miR-34c.

miRNAs can regulate gene expression by binding to the 3′ untranslated region (3′ UTR) of target mRNA. Since above we showed that miR-34c-3p expression can modulate the dissemination phenotype of *Theileria*-transformed macrophages, we looked for potential target genes that could explain the mechanism by which this is achieved. Using multiple algorithms (miRDB, miRWalk, and TargetScan), *prkar2b* was predicted to be a miR-34c-target gene in both Bos taurus and Homo sapiens. In addition, we applied another criterion requiring that the predicted seeds occur in genes whose transcriptome sequencing (RNAseq)-determined expression was low in the virulent macrophages (where miR-34c-3p levels are high) and high in the attenuated macrophages (where miR-34c-3p levels are low) (15). Combined, this identified a canonical miR-34c-3p seed sequence in the 3′ UTR of *prkar2b*, one of the 4 genes coding for regulatory subunits of PKA.

**FIG 3:**
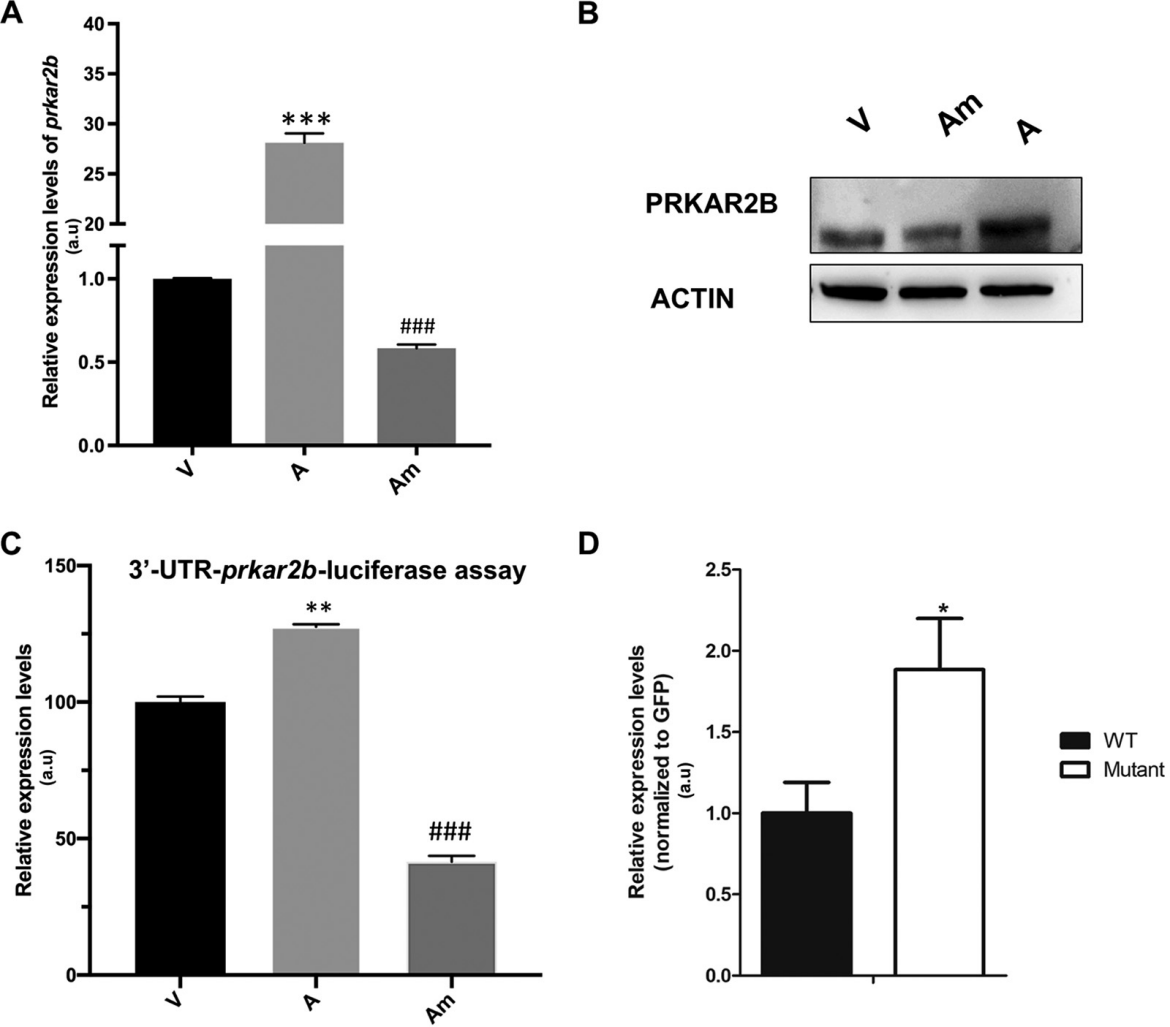
*prkar2b* is a direct miR-34c-3p-target gene. (A) Attenuated macrophages transfected with the miR-34c-3p mimic (Am) downregulate the amount of *prkar2b* transcript. (B) PRKAR2B protein levels also decrease in attenuated macrophages transfected with the miR-34c-3p mimic. (C) Reflecting greater *prkar2b* expression in attenuated macrophages, *prkar2b*-luciferase activity is higher in attenuated (A) than virulent (V) macrophages. Luciferase activity decreases when attenuated macrophages are transfected with the miR-34c-3p mimic (Am). (D) *prkar2b* mRNA levels were estimated using qRT-PCR in virulent macrophages transfected with either WT or mutant PRKAR2B expression plasmids. Mutation of potential seed sites rendered *prkar2b* transcripts resistant to miR-34c dicing.

Mammalian PKA is a heterotetrameric enzyme composed of two catalytic subunits associated with two regulatory subunits. The catalytic subunit PKA-C is bound to an inhibitory regulatory subunit (PKA-R), but upon binding of cAMP to the regulatory subunits, catalytic subunits are released to act as a serine/threonine kinase in both the cytoplasm and nucleus to phosphorylate the target proteins. PKA-C substrates include transcription factors and other proteins involved in developmental processes (22). We have previously shown that cAMP levels and PKA activity influence the dissemination potential of *Theileria*-transformed macrophages (20), and as *prkar2b* is one of four genes coding for regulatory subunits of PKA, we determined whether *prkar2b* was a bona fide miR-34c-3p target. First, the *prkar2b* mRNA levels were estimated by reverse transcription-quantitative PCR (qRT-PCR) in virulent and attenuated macrophages and following transfection of attenuated macrophages with the miR-34c-3p mimic (Fig. 3A). Consistent with being a miR-34c-3p target, *prkar2b* transcripts are more abundant in attenuated macrophages that have lower miR-34c-3p levels that increase upon transfection with the miR-34c mimic (Fig. 1A). Consequently, PRKAR2B protein levels, which were high in the attenuated macrophages, decreased when the attenuated macrophages were transfected with the miR-34c-3p mimic (Fig. 3B). To confirm that *prkar2b* was a direct target gene, its 3′ UTR, harboring the identified canonical miR-34c-3p seed sequence, was subcloned into the psiCHECK-2 vector (Promega; C8021) and transfected into attenuated macrophages, together with the miR-34c-3p mimic, and the *Renilla* luciferase activity was monitored (Fig. S1). Due to attenuated macrophages having lower levels of miR-34c-3p, they displayed greater luciferase activity than virulent macrophages (Fig. 3C, V versus A). Upon mimic treatment, the luciferase activity in the attenuated macrophages dropped significantly, confirming that *prkar2b* is a direct target of miR-34c-3p (Fig. 3C, Am). In addition, we synthesized full-length *prkar2b*, with all potential miR-34 seeds mutated (Fig. S2), and as a control, synthesized full-length wild-type (WT) *prkar2b*. The two synthesized versions of *prkar2b* were cloned with green fluorescent protein (GFP) fused to the N terminus of PRKAR2B. The level of *prkar2b* transcripts was normalized to *gfp* mRNA to compensate for any differences in transfection efficiency and expression of the 2 plasmids, and this showed that ablating the seeds rendered the *prkar2b* transcripts resistant to miR-34c-mediated dicing (Fig. 3D). Taken together, this indicates that *prkar2b* expression is regulated by variations in miR-34c-3p levels and confirms that *prkar2b* mRNA possesses a bona fide miR-34c-3p seed sequence.

10.1128/msphere.00526-22.1 FIG S1 Scheme describing the PRKR2B-3′-UTR luciferase assay. Using the Psicheck-2 vector, the predicted canonical seed sequence in the *prkar2b* 3′ UTR was inserted into the *Renilla* luciferase (hRluc) gene 3′-UTR sequence. The firefly luciferase gene (*hluc*^+^) was used as a control gene to normalize fluorescence expression. miR-34c-3p mimic was cotransfected with Psicheck-2-*prkar2b*. Luciferase activity was significantly reduced in attenuated macrophages following transfection of the miR-34c-3p mimic. Download FIG S1, TIF file, 0.5 MB. Copyright © 2023 Haidar et al. 2023 Haidar et al. https://creativecommons.org/licenses/by/4.0/ This content is distributed under the terms of the Creative Commons Attribution 4.0 International license.

10.1128/msphere.00526-22.2 FIG S2 Sequences of wild-type (WT) and mutant *prkar2b* with all 4 of the predicted noncanonical miR-34 seeds in the coding sequence mutated. The original seed sites are highlighted in yellow and the mutated sites in green. Download FIG S2, TIF file, 0.1 MB. Copyright © 2023 Haidar et al. 2023 Haidar et al. https://creativecommons.org/licenses/by/4.0/ This content is distributed under the terms of the Creative Commons Attribution 4.0 International license.

**FIG 4:**
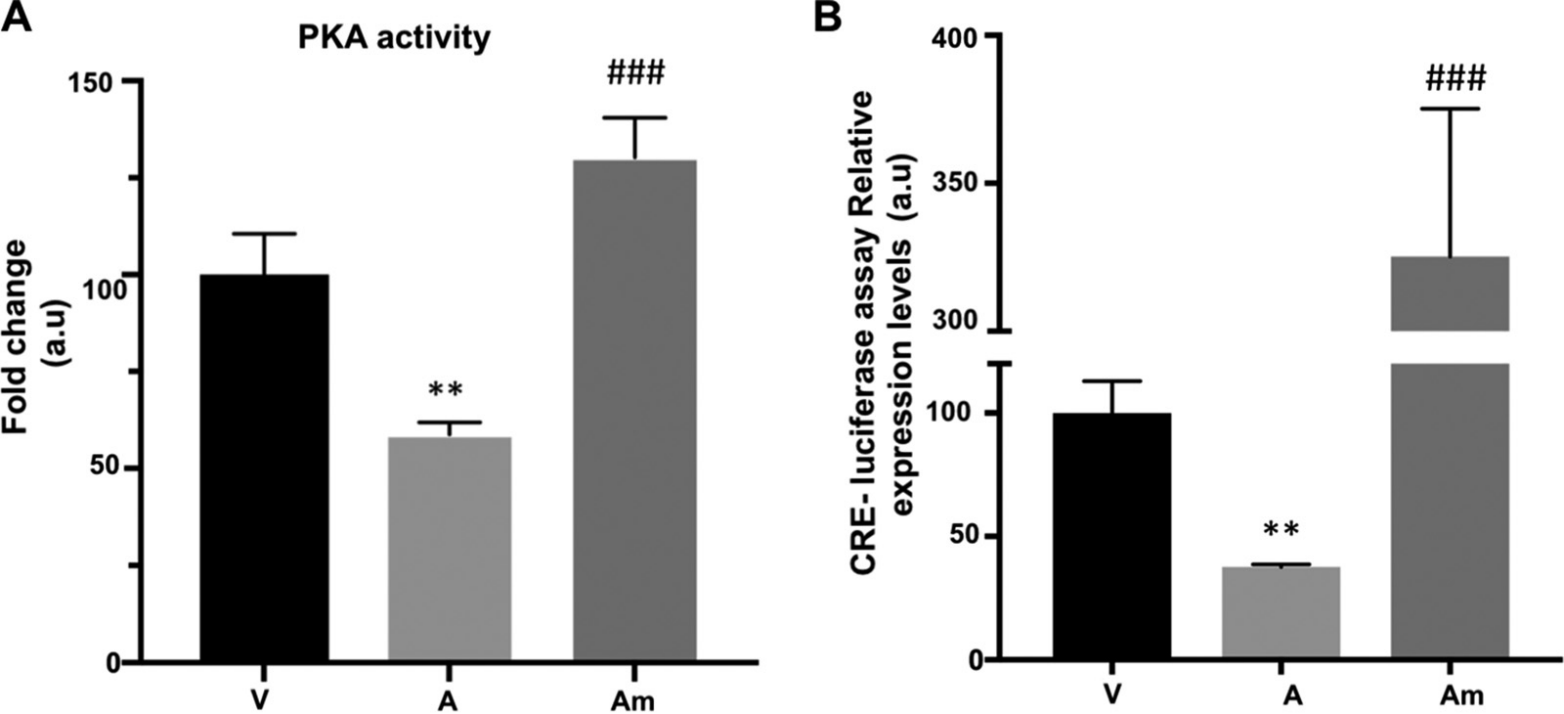
miR-34c-3p-induced drop in PRKAR2B amounts upregulates the PKA kinase activity of *T. annulata*-infected macrophages. (A) *T. annulata*-infected virulent (V) macrophages have higher PKA activity than attenuated (A) macrophages, and transfection with the miR-34c-mimic (Am) restores PKA activity in attenuated macrophages. (B) CRE-driven luciferase activity is higher in virulent than in attenuated macrophages. Transfection of attenuated macrophages with the miR-34c-mimic increases CREB transactivation of luciferase. Data are represented as mean ± SEM. *n *=* *3. ***, *P < *0.001 compared to virulent macrophages; ###, *P < *0.001 compared to attenuated macrophages.

We have previously established that dissemination of *T. annulata*-transformed macrophages is cAMP-PKA dependent (20). In Fig. 2, we showed that augmenting miR-34c levels increased the Matrigel traversal of attenuated macrophages, suggesting that miR-34c-3p dampening of PRKAR2B expression increases PKA activity and macrophage traversal. Virulent (V) macrophages display higher PKA activity compared to attenuated (A) macrophages, and transfection of attenuated macrophages with the miR-34c-3p mimic (Am) restored PKA activity to virulent levels (Fig. 4A). Thus, miR-34c-3p increases both the PKA activity and dissemination potential of *Theileria*-transformed macrophages by reducing the amounts of PRKAR2B.

PKA is known to phosphorylate the transcription factor cAMP-responsive element binding protein (CREB), so for further confirmation of the capacity of miR-34c-3p to modulate PKA activity, the transcription level of CREB was estimated by CREB Responsive Element (CRE)-driven luciferase assay. This assay relies on the use of the firefly luciferase gene under the control of CRE located upstream of the promoter, such that when PKA phosphorylates CREB, it binds CRE, which augments the expression of luciferase (Fig. S3). As expected, we found that miR-34c-3p mimic treatment induces an increase in CRE-driven luciferase activity (Fig. 4B). Taken together, this demonstrates that mammalian PKA kinase activity can be regulated independently of changes in cAMP levels via miR-34c-3p-mediated dicing of *prkar2b*.

10.1128/msphere.00526-22.3 FIG S3 Scheme explaining the principle of the CRE luciferase assay. PKA phosphorylation of the cAMP-responsive element binding protein (CREB) stimulates its binding to CRE, which promotes expression of luciferase. R2B, cAMP-dependent protein kinase A type II-beta regulatory subunit; C2B, type II-beta catalytic subunit. Download FIG S3, TIF file, 0.3 MB. Copyright © 2023 Haidar et al. 2023 Haidar et al. https://creativecommons.org/licenses/by/4.0/ This content is distributed under the terms of the Creative Commons Attribution 4.0 International license.

### miR-34c-3p regulates P. falciparum-infected erythrocyte PKA kinase activity.

**FIG 5:**
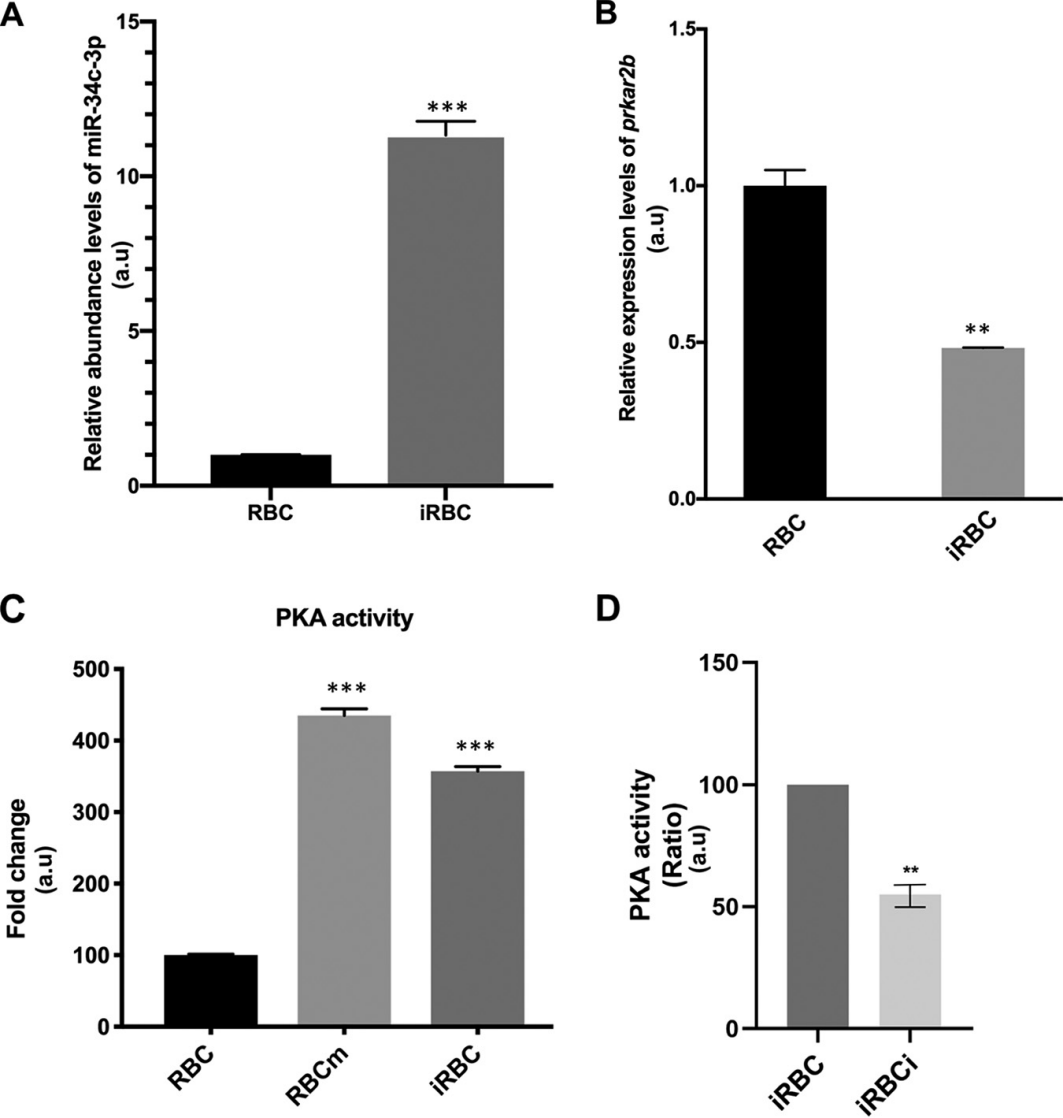
miR-34c-3p targets *prkar2b* to regulate P. falciparum-infected erythrocyte PKA activity independently of fluxes in cAMP. (A) miR-34c-3p levels drastically increase in infected red blood cells (iRBCs) compared to noninfected red blood cells (RBCs). (B) The level of *prkar2b* transcripts estimated by qRT-PCR is higher in noninfected RBCs compared to that in P. falciparum-infected RBCs. (C) Infected RBCs (dark gray column) display higher PKA activity than noninfected RBCs (black column). Transfection of noninfected RBCs with the miR-34c mimic (RBCm) leads to an increase in PKA activity (light gray column). (D) Inhibition of miR-34c-3p binding to *prkar2b* in infected RBCs (iRBCi) decreases PKA activity. Data are represented as mean ± SEM. *n* = 3. **, *P* < 0.005; ***, *P* < 0.001.

As red blood cell infection by P. falciparum has been reported to increase intraerythrocyte miR-34c-3p levels (see the supplementary files in reference 18), and given that above, we showed that changes in miR-34c-3p levels impacted the PKA activity of *T. annulata*-infected macrophages, we examined whether miR-34c-3p could regulate the PKA activity in P. falciparum-infected RBCs. First, we confirmed that infection with P. falciparum does indeed lead to increased amounts of intraerythrocyte miR-34c-3p (Fig. 5A), and then we demonstrated that this increase correlated with a reduction in the abundance of *prkar2b* mRNA (Fig. 5B) and increased PKA activity (Fig. 5C). Competitive inhibition of miR-34c-3p binding to its cognate seed sequence raised *prkar2b* transcript levels and reduced the PKA kinase activity of infected red blood cells (Fig. 5D), demonstrating that changes in PKA activity are directly due to miR-34c-3p binding to *prkar2b*.

## DISCUSSION

MicroRNA functions are possibly associated with neoblast biology, development, physiology, infection, and immunity of parasites (23). Importantly, parasite infection can alter host miRNA expression, which can favor both infection and parasite clearance. In this study, we first established that infection-induced augmentation in host cell miR-34c-3p levels reduced the expression of the *prka2b*/PRKA2B subunit, leading to increased host cell PKA kinase activity in both *Theileria*-infected leukocytes and *Plasmodium*-infected erythrocytes. First, we showed that when the miR-34c-3p seed in the 3′ UTR of *prkar2b* was fused to the luciferase gene, the amount of luciferase activity decreased when *T. annulata*-infected macrophages were cotransfected with a miR-34c-3p mimic. Then, we used two different technical approaches to further demonstrate the miR-34c-3p-mediated dicing of *prka2b*, and this was due to leukocytes being nucleate, whereas erythrocytes are anucleate. In the case of *T. annulata*-infected leukocytes, all potential miR-34c-3p seed sites in *prka2b* were mutated and the infected host leukocytes transfected with GFP-tagged plasmids encoding either wild-type or mutant *prka2b*. This showed that the loss of miR-34c-3p seed sequences significantly dampened the miR-34c-mediated dicing of *prka2b.* Since a plasmid-based transgenetic methodology is not applicable to P. falciparum-infected erythrocytes that lack a nucleus, a commercially available inhibitor of human miR-34c-3p binding to its seed sequence was used to again demonstrate that mammalian *prka2b* is a direct miR-34c-3p target. We did not treat virulent *T. annulata*-infected leukocytes with the human miR-34c-3p inhibitor due to differences in the cognate seed sequence present in the 3′ UTR of human (AUCACUA) and bovine (GUGACGG) *pkar2b*. Moreover, although infection-induced miR-34c-3p levels are high in virulent and low in attenuated macrophages, we did not treat virulent macrophages with a custom-made bovine-specific competitive inhibitor of miR34c-3p. The reason was that in virulent macrophages, TGF-β, PGE2 signaling, and cAMP levels are all high and contribute to the transformed phenotype (20), potentially masking any antioncogenic effect of inhibiting miR-34c-3p-mediated dicing. However, the levels of TGF-β, PGE2, and cAMP are all significantly lower in attenuated macrophages (20), so they were treated with the mimic of miR34c-3p to reveal its oncogenic potential.

We demonstrated that miR-34c-3p diminishes the amounts of PRKAR2B, subsequently leading to enhanced PKA activity and CREB transactivation, both of which we have shown are important contributors to *Theileria*-transformed macrophage dissemination. Previously, we described how TGF-β secreted by *T. annulata*-transformed macrophages contributes to sustaining PKA activity in virulent macrophages via PGE2 engagement of EP4 to augment levels of cAMP (20). Now, we provide an infection-induced epigenetic mechanism for regulating mammalian PKA activity independent of fluxes in cAMP. *T. annulata* infection and transformation of bovine macrophages target mammalian PKA activity in 3 different ways: (i) by raising cAMP levels (20); (ii) by suppressing the endogenous inhibitor PKIG (20); and (iii) by miR-34c-3p-mediated dampening of PRKAR2B subunit expression. This three-pronged “attack” underscores the key role PKA plays in the dissemination of *T. annulata*-transformed macrophages.

**FIG 6:**
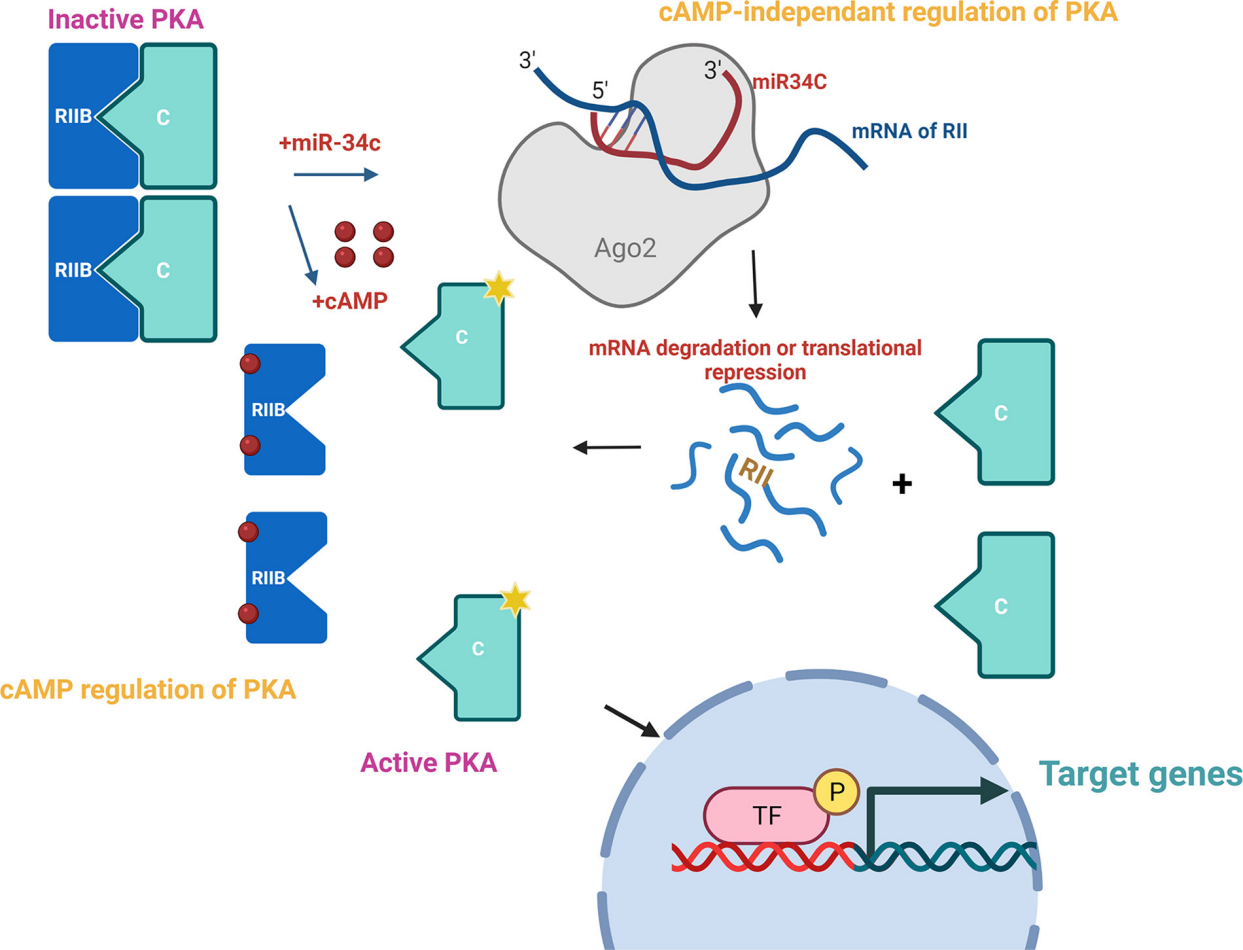
Model proposing how miR-34c-3p alters PKA kinase activity by targeting PRKAR2B, independent of fluxes in cAMP. PKA is a heterodimer composed of two catalytic subunits bound to two regulatory subunits. Classical activation of PKA occurs when cAMP binds to regulatory subunits, causing a conformational change that liberates catalytic subunits from an inactive R2/C2 complex. miR-34c-3p can regulate PKA catalytic activity independently of cAMP by dicing *prkar2b*, leading to reduced PRKAR2B expression and greater PKA catalytic activity toward target proteins in the nucleus and cytosol.

In addition, we provide evidence that miR-34c-3p also targets mammalian *prkar2b* in P. falciparum-infected erythrocytes. First, we confirmed a previous report (18) that miR-34c-3p levels are higher in RBCs infected with P. falciparum than in noninfected erythrocytes. Although red blood cell miR-34c-3p targets were not previously characterized (18), we now show that the amount of *prkar2b* mRNA in infected erythrocytes increases upon treatment with an antagonist that competitively inhibits miR-34c-3p binding to its seed sequences to increase the *prkar2b* abundance and reduce PKA activity. Overall, our results suggest that miR-34c-3p mimics and antagonists could have therapeutic use in the treatment of different diseases caused by intracellular parasites, and in Fig. 6, we present a generalized schematic representing how miR-34c-3p-mediated dicing of *prkar2b* reduces PRKAR2B amounts to modulate the PKA kinase activity independent of fluxes in cAMP in *Theileria*-infected leukocytes and *Plasmodium*-infected erythrocytes.

## MATERIALS AND METHODS

### Cell culture.

The cells used in this study were *T. annulata*-transformed Ode macrophages; the virulent macrophages used correspond to passage 62 and the attenuated macrophages to passage 364 (21). The noninfected BL20 and *T. annulata*-infected BL20 (TBL20) cells used were previously characterized (24, 25). All cells were incubated at 37°C under 5% CO_2_ in RPMI medium supplemented with 10% fetal bovine serum (FBS), 2 mM l-glutamine, 100 U penicillin, 0.1 mg/mL streptomycin, HEPES, and 5% 2-mercaptoethanol for BL20 and TBL20.

### Malaria parasite culturing.

P. falciparum 3D7 parasites were cultured in RPMI 1640 medium (51800-035; Life Technologies) supplemented with 0.5% (wt/vol) Albumax II (11021-29), 200 μM hypoxanthine, and 20 μg/mL gentamicin (complete RPMI 1640 medium) in human erythrocytes at a hematocrit between 2.5% and 5%. Parasite cultures were maintained under low oxygen pressure (1% O_2_, 3% CO_2_, 96% N_2_) at 37°C.

### RNA extractions.

Total RNA was isolated from *Theileria*-infected leukocytes using the Direct-zol RNA kit (R2070; Zymo Research), and the total RNA designated for miRNA experiments was extracted using the mirVana miRNA isolation kit (Thermo Fisher) according to the manufacturer’s instructions. The quality of the extracted RNA was verified using a Bioanalyzer 2100 system, and quantification was carried out using Qubit (catalog number Q10210; Invitrogen).

### qRT-PCR for miRNAs.

The RNA extracted with the mirVana kit was used. cDNA was synthesized using the TaqMan microRNA RT kit (Applied Biosystems) following the manufacturer’s instructions. A total of 10 ng of RNA was used in the reverse transcription reaction with miRNA-specific primers. The real-time reactions were performed in a 7500 high-throughput (HT) fast real-time PCR system (Applied Biosystems). Data were analyzed using the comparative threshold cycle (2^−ΔΔCT^) method. The threshold cycle (*C_T_*) values for the selected miRNA targets were subtracted from the *C_T_* values of the endogenous small noncoding RNA control RNU6B (control miRNA assay; Applied Biosystems).

### qRT-PCR for mRNA.

Total RNA was reverse transcribed using the high-capacity cDNA reverse transcription kit (Applied Biosystems; catalog number 4368814), as follows: 1 μg total RNA, 2 μL RT buffer, 0.8 μL 100 mM deoxynucleoside triphosphate (dNTP) mix, 2.0 μL 10× random primers, 1 μL MultiScribe reverse transcriptase, and nuclease-free water to a final volume of 20 μL. The reaction mixture was incubated for 10 min at 25°C and then 2 h at 37°C; subsequently, the enzyme was inactivated at 85°C for 5 min. Real-time PCR was performed in a 20-μL reaction mixture containing a cDNA template, 10 μL 2× fast SYBR green master mix, and 500 nM forward and reverse primers. The reaction was run on the 7500 HT fast real-time PCR system (Applied Biosystems). Expression of *gapdh*, *hprt1*, and beta-actin was used as the measure of housekeeping gene expression, and the results were analyzed using the 2^−ΔΔCT^ method. The error bars represent the standard error of the mean (SEM) of 3 biological replicates.

### Western blotting.

Cells were harvested and extracted using 1× mammalian lysis buffer (ab179835; Abcam) supplemented with protease and phosphatase inhibitor cocktail (78440; Thermo Fisher). The protein concentration was determined using the Bradford protein assay. Cell lysates were subjected to Western blot analysis using conventional SDS/PAGE and protein transfer to nitrocellulose filters (Protran, Whatman). The membrane was blocked using 5% nonfat milk-TBST (Tris-buffered saline with Tween 20) (for anti-PRKAR2B and anti-GAPDH), or 3% nonfat milk-PBST (phosphate-buffered saline with Tween 20) (for anti-actin antibody) overnight at 4°C. The antibodies used for immunoblotting were as follows: rabbit polyclonal antibody anti-GAPDH (ABS16; Merck Millipore), mouse monoclonal antibody anti-PRKAR2B (610625; BD Transduction Laboratories), and goat polyclonal antibody anti-actin (Santa Cruz Biotechnologies; I-19). After washing, proteins were visualized using enhanced chemiluminescence (ECL) Western blotting detection reagents (Thermo Scientific) with a fusion instrument. The beta-actin was used as a loading control throughout all experiments.

### Fluorescence-activated cell sorting of GFP-PRKAR2B-expressing cells.

Wild-type *prkar2b* subunit of bovine PKA and mutant *prkar2b* (harboring mutations that ablate all potential miR-34c seed sites) were synthesized (GenScript) and cloned into pmaxGFP vector (Lonza). Virulent *T. annulata*-transformed macrophages were transfected with plasmids expressing wild-type or mutant PRKAR2B with GFP fused to the N termini. The transfection rate (efficiency) was measured, and in 3 independent transfections, the efficiency averaged 19%. Therefore, to obtain a high percentage of transfected cells, 24 h posttransfection, GFP-expressing cells were separated using fluorescence-activated cell sorting (FACS) with the MoFlo Astrios instrument (Beckman). Following sorting, total RNA was prepared as described above.

### Matrigel chamber assay.

The dissemination capacity of the Ode macrophages was assessed *in vitro* using Matrigel migration chambers, as described previously (26). The culture coat medium BME (basement membrane extract) 96-well assay was performed according to the Cultrex instructions (catalog number 3482-096-K). After 24 h of incubation at 37°C, each well of the top chamber was washed once in the buffer. The top chamber was replaced onto the receiver plate. One hundred microliters of cell dissociation solution-calcein AM (acetoxymethyl) was added to the bottom chamber of each well, and the mixtures were incubated at 37°C for 1 h with fluorescently labeled cells to dissociate the cells from the membrane before reading at 485 nm excitation and 520 nm emission wavelengths, using the same parameters as for the standard curve.

### Transfection.

Macrophages were transfected by electroporation using the Nucleofector system (Amaxa Biosystems). A total of 5 × 10^5^ cells were suspended in 100 μL of Nucleofector solution mixed with 400 pM of mimic of miR-34c-3p and subjected to electroporation using the cell line-specific program T-O17 for *Theileria*-infected macrophages. After transfection, cells were suspended in a fresh complete medium and incubated at 37°C under 5% CO_2_ for 48 h.

### Synchronization of P. falciparum-infected red blood cell culture and pharmacological inhibition or stimulation of miR-34c-3p.

Parasites were cultured to at least 10% parasitemia in T-75 flasks containing 25 mL medium at 2% hematocrit. The parasites were synchronized first at the ring stage with two rounds of 5% sorbitol. When most of the parasites had matured to schizonts, the culture was loaded onto a 70% Percoll cushion and centrifuged for 10 min at 800 × *g*. Schizonts from the top of the 70% Percoll cushion were collected, washed two times with complete medium, and resuspended in complete medium. Parasitemia was adjusted to 0.2 to 0.5% and 2% hematocrit, and the culture was distributed in triplicate in 6-well plates with various treatments: 400 pM of the mimic (HMI0513-5NMOL; Sigma) or 400 pM of the inhibitor (catalog number 4464084; Ambion from Thermo Fisher). The culture was incubated at 37°C under a 5% CO_2_/3% O_2_/balanced N_2_ gas mixture for 96 h. To follow the growth, blood smears were prepared and stained with Giemsa solution (Sigma).

### CREB luciferase assay.

After transfection, the cells were suspended in fresh complete medium, incubated at 37°C and 5% CO_2_ for 24 h, and lysed after 48 h. According to the manufacturer’s instructions, the luciferase and β-galactosidase activities were measured using the dual light assay system (Life Technologies) and Centro LB 960 luminometer (Berthold).

### Measurement of PKA activity.

*Theileria*-infected macrophages were transfected with the specific mimic of miR-34c-3p. Samples were then collected, centrifuged at 1,500 rpm for 5 min, and lysed using the recommended lysis buffer (20 mM MOPS, 50 mM β-glycerol phosphate, 50 mM sodium fluoride, 1 mM sodium vanadate, 5 mM EGTA, 2 mM EDTA, 1% NP-40, 1 mM dithiothreitol [DTT], 1 mM benzamidine, 1 mM phenylmethylsulfonyl fluoride [PMSF], and 10 μg/mL leupeptin and aprotinin). For permeabilization of P. falciparum-infected red blood cells, Streptolysin O (SLO; Sigma) was first titrated by resuspending SLO (25,000 units) in 5 mL of PBS (10 mM sodium phosphate buffer and 145 mM NaCl, pH 7.4) containing 0.1% bovine serum albumin (BSA) and activating it by incubation with 5 to 10 mM dithiothreitol at 37°C for 2 h. iRBCs were resuspended in 200 μL of RPMI medium containing 3 to 4 hemolytic units of SLO, resulting in hemoglobin release of more than 98%. Following permeabilization with SLO, the cells were centrifuged and washed with 200 μL RPMI. P. falciparum-infected erythrocyte pellets were lysed using the recommended buffer, and the lysates were cleared by centrifugation (15,000 rpm for 15 min at 4°C); the total amount of proteins in the supernatant was measured using a Bio-Rad protein assay, based on the method of Bradford, using BSA as a standard. PKA activity was measured using an enzyme-linked immunosorbent assay (ELISA) kit (PKA kinase activity kit, 139435; Abcam) according to the manufacturer’s instructions.

### Statistical analysis.

Data were analyzed using Student’s two-tailed *t* tests, with 3 biological replicates for each experiment. All values are expressed as the mean ± SEM. In each figure, the following symbols represent the respective *P* value ranges: *, *P* < 0.05; **, *P* < 0.005; and ***, *P* < 0.001.
