# Supplementary figures and images for: miR-34c-3p Regulates Protein Kinase A Activity Independent of cAMP by Dicing prkar2b Transcripts in Theileria annulata-Infected Leukocytes
Source: mSphere. 2023 Feb 27;8(2):e00526-22. doi: 10.1128/msphere.00526-22 (PMC10117149; doi:10.1128/msphere.00526-22)

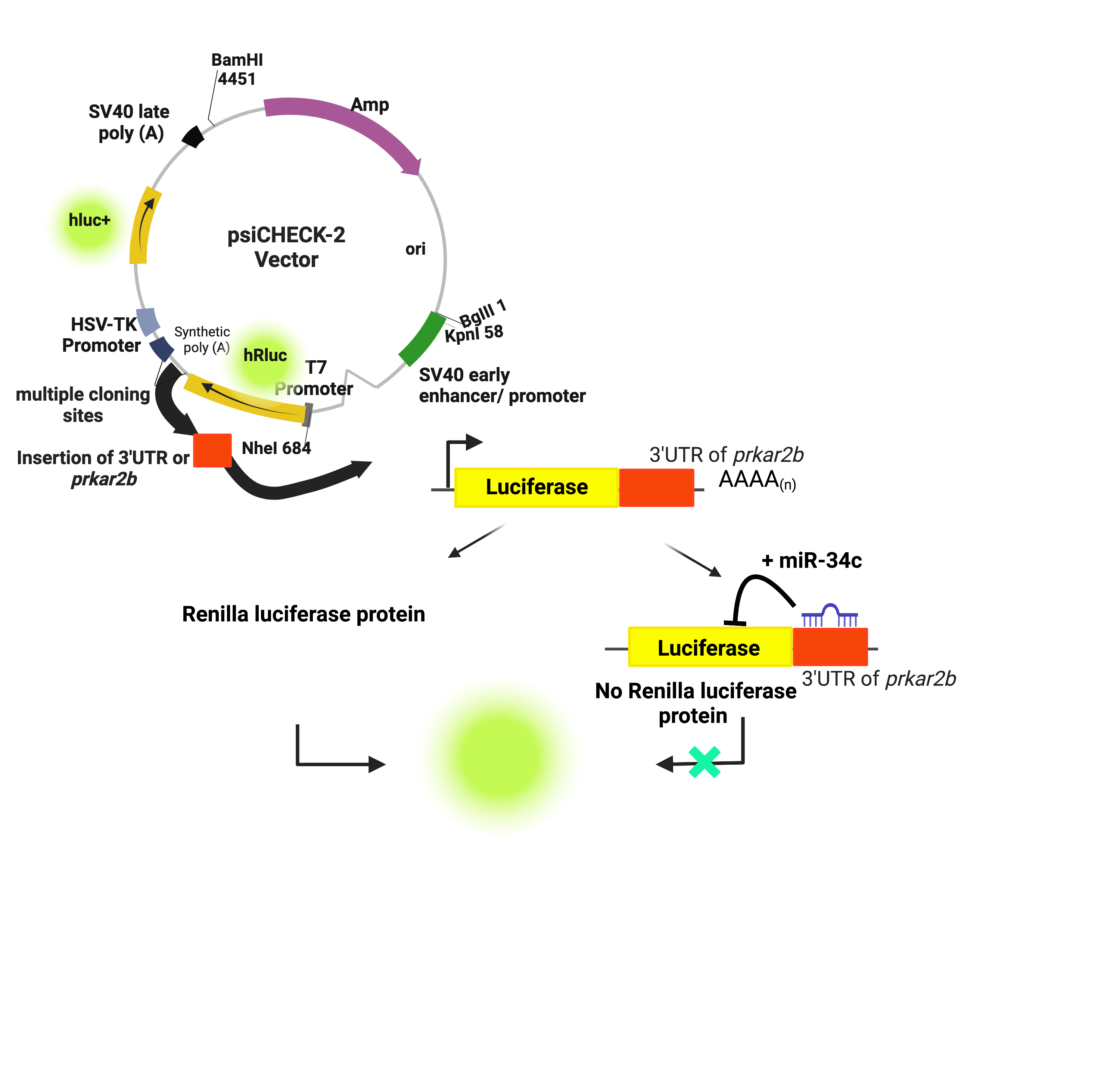

Supplement: FIG S1 [file msphere.00526-22-s0001.tif]

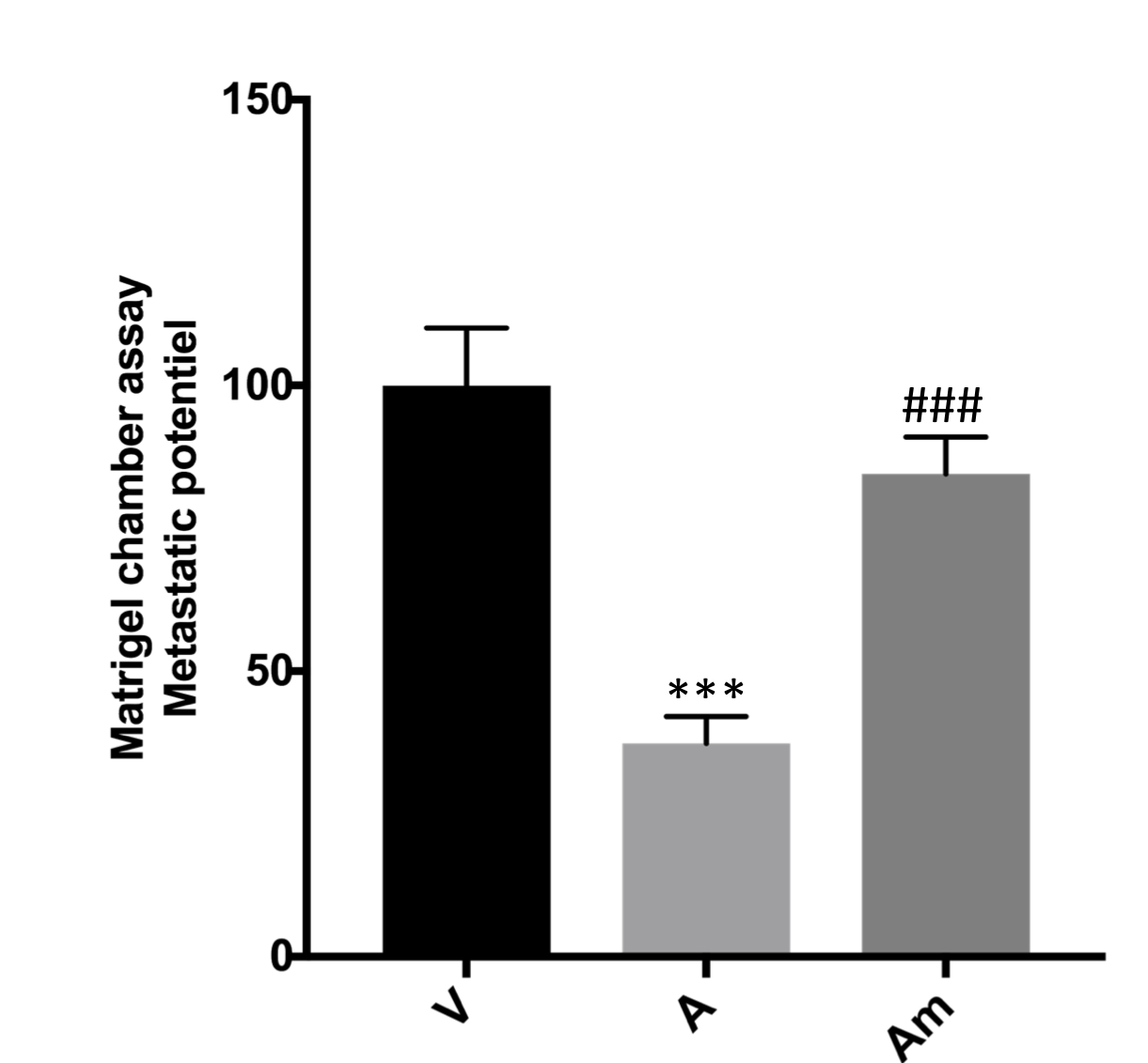

Supplement: FIG S2 [file msphere.00526-22-s0002.tif]

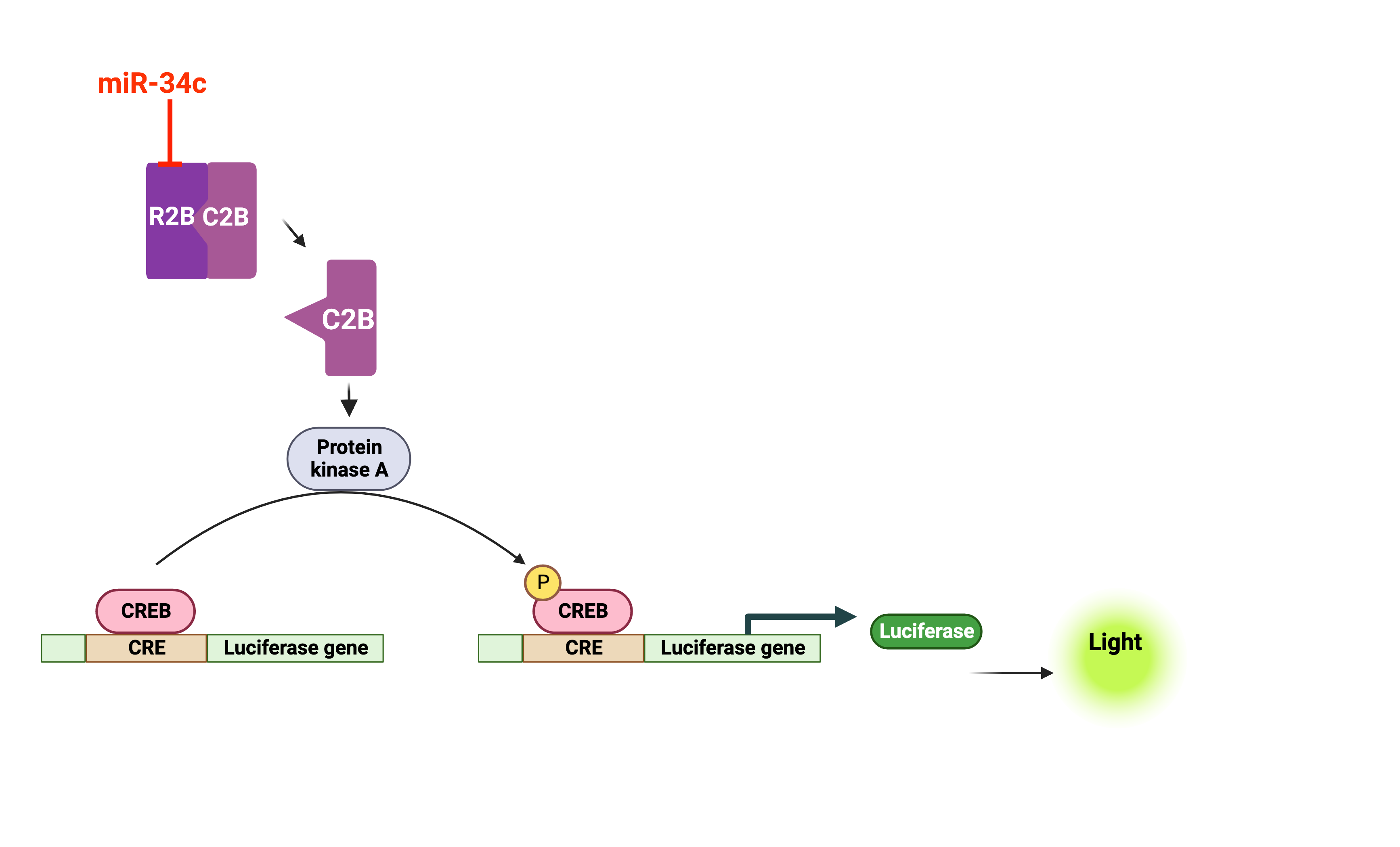

Supplement: FIG S3 [file msphere.00526-22-s0003.tif]
